# Episodic, transient systemic acidosis delays evolution of the malignant phenotype: Possible mechanism for cancer prevention by increased physical activity

**DOI:** 10.1186/1745-6150-5-22

**Published:** 2010-04-20

**Authors:** Kieran Smallbone, Philip K Maini, Robert A Gatenby

**Affiliations:** 1 Manchester Centre for Integrative Systems Biology, Manchester Interdisciplinary Biocentre, 131 Princess Street, Manchester, M1 7DN, UK; 2 School of Mathematics, University of Manchester, Oxford Road, Manchester M13 9PL, UK; 3 Centre for Mathematical Biology, Mathematical Institute, 24-29 St Giles', Oxford, OX1 3LB, UK; 4 Oxford Centre for Integrative Systems Biology, Dept. of Biochemistry, South Parks Road, Oxford, OX1 3QU, UK; 5 Moffitt Cancer Center, 12902 Magnolia Drive, Tampa, FL 33612, USA

## Abstract

**Background:**

The transition from premalignant to invasive tumour growth is a prolonged multistep process governed by phenotypic adaptation to changing microenvironmental selection pressures. Cancer prevention strategies are required to interrupt or delay somatic evolution of the malignant invasive phenotype. Empirical studies have consistently demonstrated that increased physical activity is highly effective in reducing the risk of breast cancer but the mechanism is unknown.

**Results:**

Here we propose the hypothesis that exercise-induced transient systemic acidosis will alter the *in situ *tumour microenvironment and delay tumour adaptation to regional hypoxia and acidosis in the later stages of carcinogenesis. We test this hypothesis using a hybrid cellular automaton approach. This model has been previously applied to somatic evolution on epithelial surfaces and demonstrated three phases of somatic evolution, with cancer cells escaping in turn from the constraints of limited space, nutrient supply and waste removal. In this paper we extend the model to test our hypothesis that transient systemic acidosis is sufficient to arrest, or at least delay, transition from *in situ *to invasive cancer.

**Conclusions:**

Model simulations demonstrate that repeated episodes of transient systemic acidosis will interrupt critical evolutionary steps in the later stages of carcinogenesis resulting in substantial delay in the evolution to the invasive phenotype. Our results suggest transient systemic acidosis may mediate the observed reduction in cancer risk associated with increased physical activity.

**Reviewers:**

This article was reviewed by Natalia Komarova (nominated by Marek Kimmel), Heiko Enderling (nominated by Marek Kimmel), Mark Little (nominated by Marek Kimmel) and Yang Kuang.

## Background

There is accumulating evidence that regular physical activity is an effective cancer prevention strategy. Friedenrich and Orenstein [1] recently reviewed over 170 epidemiological studies and concluded that evidence for decreased cancer risk with increased physical activity was convincing for breast and colon cancer, probable for prostate cancer, possible for lung cancer and unknown for other sites. In breast cancer, physical activity has been well documented to reduce cancer risk by almost 50% [2-4] in a wide range of populations but the mechanism of protection remains unknown. Exercise-induced alteration of ovarian function with subsequent alteration of circulating hormones has been proposed. However, a recent study demonstrated that exercise also reduces breast cancer risk in post-menopausal women [5]. Other proposed mechanisms include weight reduction and enhanced immunological activity [1-3].

The transition from normal tissue to cancer is termed carcinogenesis and is a prolonged, multi-step process. Models of carcinogenesis typically focus on heritable changes that alter tumour survival, proliferation, senescence and apoptosis. Recently, it has been proposed that regional hypoxia and acidosis also play critical roles in the final stages of carcinogenesis. Using mathematical models, *in vitro *experiments, and clinical observations, it has been shown that proliferation of *in situ *cancers carries the cells further away from the basement membrane and, therefore, increasingly distant from the underlying blood supply [6-8]. The resulting reaction-diffusion kinetics results in marked hypoxia in cells more than 5 or 10 cell layers from the basement membrane [9]. The consequent upregulation of glycolysis combined with long diffusion distances for metabolites produces marked acidosis in the hypoxic regions [10]. It has been proposed that adaptation to hypoxia is critical in emergence of the malignant phenotype because the combination of adaptations with upregulation of glycolytic metabolism and resistance to acid-mediated toxicity confers a profound proliferative advantage [9]. Specifically, this population produces an acidic environment through upregulated glycolysis that is toxic to competing populations but not to its own cells. Clinical observations in both breast [7] and cervical cancers [11] have found evidence that successful adaptation to the hypoxia-glycolysis-acidosis sequence in the microenvironment is crucial during carcinogenesis.

Here we use these results to propose an alternative mechanism for the protective effects of physical activity in cancer prevention. We hypothesise that exercise produces toxicity within *in situ *cancers through transient decreases in serum pH and, by doing so, delays or prevents somatic evolution of a malignant phenotype. To support this hypothesis, we note that there is clear evidence that even moderate exercise transiently decreases serum pH typically from its normal value of about 7.4 to about 7.25. With moderate exercise for one hour, the diminished pH level generally persists for about an hour [12,13]. Based on this, our detailed hypothesis is that decreased blood pH will also transiently cause significant further decrease in extracellular pH (pH_e_) in the already acidic regions within *in situ *cancers. This abrupt increase in acid concentrations will result in tumour cell death and interrupt the adaptive mechanisms necessary for subsequent evolution to the malignant phenotype.

The hypothesis generates a number of initial questions:

• Do transient decreases of blood pH decrease the pH_e _of *in situ *cancers?

• Are the intra-tumoral pH_e _effects linearly dependent on the blood pH value or are there threshold levels?

• If the pH_e _is decreased, is this change sufficient to induce death in some of the tumour cells?

• Will repeated transient episodes stop or slow the somatic evolution process and thus delay or prevent emergence of invasive cancer?

• If increased physical activity does alter *in situ *tumour growth and evolution, what is the optimal level of activity (exercise intensity and frequency)?

To address some of these questions, we frame our hypothesis using a mathematical model of the evolutionary dynamics and changing microenvironmental selection conditions in *in situ *cancer. As we have previously demonstrated, *in situ *cancers arise from epithelial surfaces and, as a result, grow into the ductal lumen and away from the underlying basement membrane. Because the blood vessels remain on the opposite side of the membrane, substrate and cellular metabolites must diffuse over increasing distances to reach the growing cell layer that is farthest from the basement membrane. For this reason, models of carcinoma *in situ *must include regional variations in oxygen, glucose and pH_e_. In turn, these microenvironmental conditions produce differing selection forces resulting in regional variations in the evolved tumour phenotype. This system presents a modelling challenge. Continuous (partial differential equation) models are well suited to modelling the diffusion-reaction kinetics that produce regional variations in substrate and metabolite concentrations. On the other hand individual-based models such as cellular automata (CA) are more appropriate when the evolutionary dynamics of individual cells must be considered. However, traditional CA methods lack the ability to deal with continuously varying elements such as substrate diffusion and utilisation. Thus, hybrid CA have been developed to investigate early cancer development [14-16]. In this paper we extend a hybrid CA we [17] have previously employed to examine phenotypic evolution and microenvironmental conditions in *in situ *cancers under conditions in which systemic pH varies.

## Methods

Automaton dynamics are described in detail elsewhere [17], but we briefly describe them here for completeness. The two-dimensional model is composed of an *M *× *N *array of automaton elements with a specific rule-set governing their evolution, as well as glucose (*g*), oxygen (*c*) and H^+ ^(*h*) fields, each satisfying reaction-diffusion equations. A two-dimensional automaton is used as we focus on growth away from the basement membrane, rather than along the duct. In the model we reflect the avascular geometry of premalignant epithelia by assuming that one edge of the array represents the basement membrane.

We consider the selective pressures placed on a number of different possible tumour phenotypes. Initially, the automaton consists of a single layer of normal epithelial tissue. As well as proliferation and death, these cells may randomly undergo three possible heritable changes, either through mutations or epigenetic changes such as alterations in the methylation patterns of promoters. The cells may become hyperplastic (allowing growth away from the basement membrane), glycolytic (increasing their rate of glucose uptake and utilisation) or acid-resistant (requiring a lower extracellular pH to induce toxicity). These three changes give rise to 2^3 ^= 8 different phenotype combinations, and thus eight competing cellular populations.

### Cellular metabolism

Suppose that the cell consumes glucose and oxygen at rates *ϕ*_*g *_and *ϕ*_*c*_, respectively, and that they are used to produce ATP and H^+ ^at rates *ϕ*_*a *_and *ϕ*_*h*_, respectively. In non-dimensional form, we have(1)

subject to the condition *ϕ*_*g *_≥ *c*. As a default value, we assume *k *= 10, i.e. that glycolytic cells demonstrate a ten-fold increase in glucose consumption.

### Metabolite profiles

After each automaton generation, the known rates of metabolite consumption and production for each cell are used to calculate the corresponding metabolite profiles. Note that metabolite diffusion time-scales (~minutes) are much shorter than the cellular proliferation time-scale (~days), and thus we may assume that metabolites are in equilibrium at all times. Assuming that diffusion is the primary method for metabolite movement within the tissue, profiles are given in non-dimensional form by(5)

which may be solved on the square grid using a finite difference approximation. As boundary conditions, we assume zero flux at the edge furthest from the basement membrane (as there are no sources or sinks beyond this point), and periodic boundary conditions at the two sides. At the membrane, we assume glucose and oxygen are fixed at their normal levels *g*_0, *j *_= *c*_0, *j *_= 1 (as the stroma is well-vascularised); H^+ ^are also fixed, *h*_0, *j *_= *h*_*X*_, where the parameter *h*_*X *_reflects the level of systemic acidosis.

### Cell dynamics

Cells may proliferate, adapt or die, and cells with different phenotypic patterns respond to the microenvironmental pressures in different ways. As such, competition is incorporated into the model: for a new population to progress and grow, it must successfully compete for space and resources with existing populations. The rules governing the evolution of the automaton elements are as follows:

• If the amount of ATP produced by a cell *ϕ*_*a *_falls below a critical threshold value, *a*_0_, it dies, and the element becomes empty; *a*_0 _represents the level of ATP required for normal cellular maintenance.

• The local H^+ ^level may also induce cellular death, with probability *p*_dea_, defined by(8)

where *h*_*N *_<*h*_*T*_. Thus the probability of cell death increases with acidity, and the cell will always die if the H^+ ^level is greater than *h*_*N *_or *h*_*T*_, dependent on the cell type under consideration.

• If the cell is not attached to the basement membrane, and is not hyperplastic, it dies.

• If the cell does not die through any of the mechanisms above, it either attempts to divide, with probability *p*_div_, or becomes quiescent. The probability of division is a function of the cellular ATP production(9)

Hence we assume that the probability of division is proportional to the ATP generated that is not needed for maintenance. If there is more than one neighbouring empty space, the new cell goes to the element with the largest oxygen concentration (following [14]).

• If a cell divides, each of the two daughter cells has probability *p*_a _of randomly acquiring one of the three heritable characteristics (hyperplasia, glycolysis and acid-resistance). In order to avoid bias in the model, we assume these changes are reversible. For example, a cell displaying constitutive up-regulation of glycolysis may revert to normal glucose metabolism; if this metabolism is most appropriate for the current microenvironmental conditions, the cell will successfully compete for resources with its neighbours.

### Transient acidosis

When investigating transient acidosis, each time-step is split into two parts: a time *τ *spent at high acidity *h*_*X*_, followed by time 1 - *τ *at normal acidity *h*_*X *_= 0. Letting *p*_0 _denote the probability of death *p*_dea_, division *p*_div_, or mutation *p*_a _during one time unit (as defined in the previous section), the corresponding probability *p *of occurrence during time *τ *∈ [0, 1] is reduced according to(10)

Throughout the model formulation, we assume that various processes follow simple, linear dynamics. It can be argued that these assumptions are too unrealistic to represent complex biological phenomena such as these. However, these processes are poorly understood and, as a first approximation, an assumption of linearity is sufficient to capture qualitatively equivalent monotonic behaviour.

## Results

**Figure 1:**
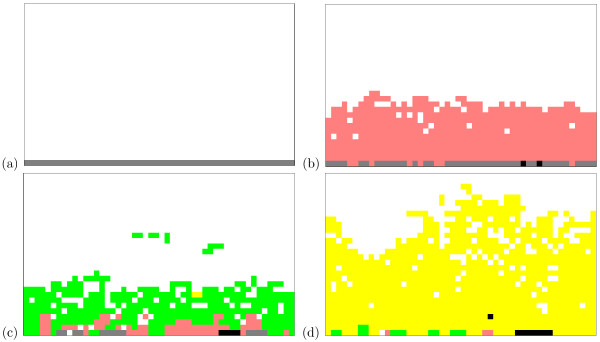
**Automaton evolution**. (Reproduced from [17].) The temporal evolution of a typical cellular automaton after (a) *t *= 0, (b) *t *= 100, (c) *t *= 250 and (d) *t *= 300 generations. Shown are normal epithelial (grey), hyperplastic (pink), hyperplastic-glycolytic (green) and hyperplastic-glycolytic-acid-resistant (yellow) cells. Cells with other phenotypic patterns are shown as black.

We apply the procedures as set out in the previous section. For a list of parameter values used, see Table 1. Consider first the model under normal serum pH (*h*_*X *_= 0), as described in previous work [17]. Fig. 1 shows the temporal evolution of a typical cellular automaton. Initially, normal epithelial cells (grey) line the basement membrane (a). Acquisition of the hyperplastic phenotype (pink) allows growth away from the membrane towards the oxygen diffusion limit (b). Beyond this point, cells cannot exist as the oxygen levels are insufficient to meet cellular ATP demands. This drives adaptation to a glycolytic phenotype (green), less reliant on oxygen for ATP production (c). The increased ATP levels within glycolytic cells give a competitive advantage over the existing population, thus glycolytic cells dominate the system. Note, however, that the total number of cells within the system has decreased; the increased reliance on glycolysis has resulted in higher levels of acidity, in turn inducing cell death. Further adaptation occurs to an acid-resistant phenotype (d). Increased use of glycolysis allows growth well beyond the oxygen diffusion limit, whilst the cells are more resistant to the resulting acidosis.

**Table 1:** List of non-dimensional parameter values.

Parameter	Value
*N*	50
*n*	5.6 × 10^-2^
*k*	10
*d*_*g*_	1.3 × 10^2^
*d*_*c*_	5
*a*_0_	0.1
*h*_*N*_	9.3 × 10^2^
*h*_*T*_	8.6 × 10^3^
*p*_a_	10^-3^
*h*_*X*_	0 or 400

Taken from an earlier work [17], in which the dimensional analogues, references and justifications are also presented.

It is interesting to note that throughout the simulations performed, the heritable changes within the dominant population are accumulated in this same order. Within our model, the underlying environmental selection parameters drive the cells to always follow this adaptive pathway - escaping in turn from the constraints of limited proliferation (hyperplasia), substrate availability (glycolysis) and waste removal (acid-resistance). The same order of progression occurs despite allowing phenotypic reversibility within our model. This means mutations are not a necessary mechanism for phenotypic variation within tumour tissue; rather the model demonstrates that reversible, epigenetic changes are sufficient to drive global change. Of course reversibility in not necessary to observe this adaptation; if irreversible, we would see the same phenotype emerge on a slightly shorter time-scale.

We now move on to investigate the effect of altering serum pH. In order to examine the effects of parameter changes on system dynamics, we define a measure of the 'fitness' of a specific parameter set. Let 'invasive' be used to describe cells displaying all three heritable changes - hyperplasia, glycolysis and acid resistance. For a particular automaton, let *T *denote the number of generations after which 95% of the cells in the system display the invasive phenotype; thus *T *is representative of the amount of time taken for full carcinogenesis to occur. Now let the development rate *R *= *T *^-1^, where we take *R *= 0 if *T *≥ 5000 (equivalent to approximately 20 years) - i.e. assume no carcinogenesis occurs. Automata with a higher value of *R *proceed more quickly through the carcinogenesis pathway.

**Figure 2:**
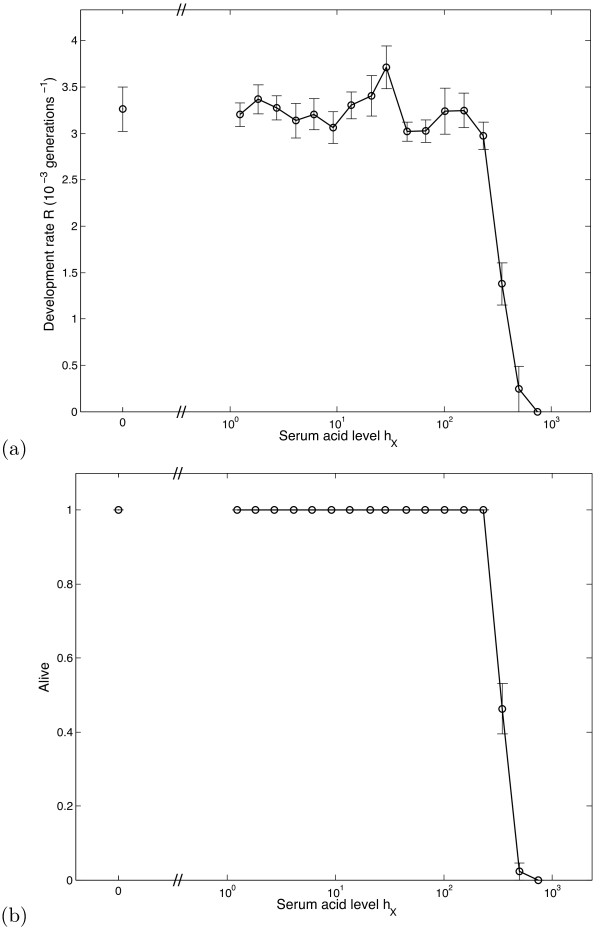
**Effect of sustained acidosis**. (a) Variation in the development rate *R *with serum acid level *h*_*X *_(plotted on a log scale). Each data point is the mean value of R calculated over 50 simulations, whilst the accompanying error bars show the standard errors of these means. (b) Variation in epithelium survival with *h*_*X*_.

In Fig. 2(a) we see how the development rate *R *varies with changes in serum acidity *h*_*X*_. We vary the external acid levels from *h*_*X *_= 0 (normal) to *h*_*X *_~1000, equivalent to pH 6.8, corresponding to the threshold for normal cell survival [16]. Decreases in pH_e _of this magnitude have been observed in various human cancers [18]. Note that a log scale is used. Development rate *R *remains fairly constant until *h ~ *100 (a drop of around 0.1 pH units), when a marked decrease is observed. Looking further however, we see (Fig. 2(b)) that this result follows simply because the harsher conditions lead to death of the entire epithelium; normal cells die out before having the opportunity to turn cancerous.

**Figure 3:**
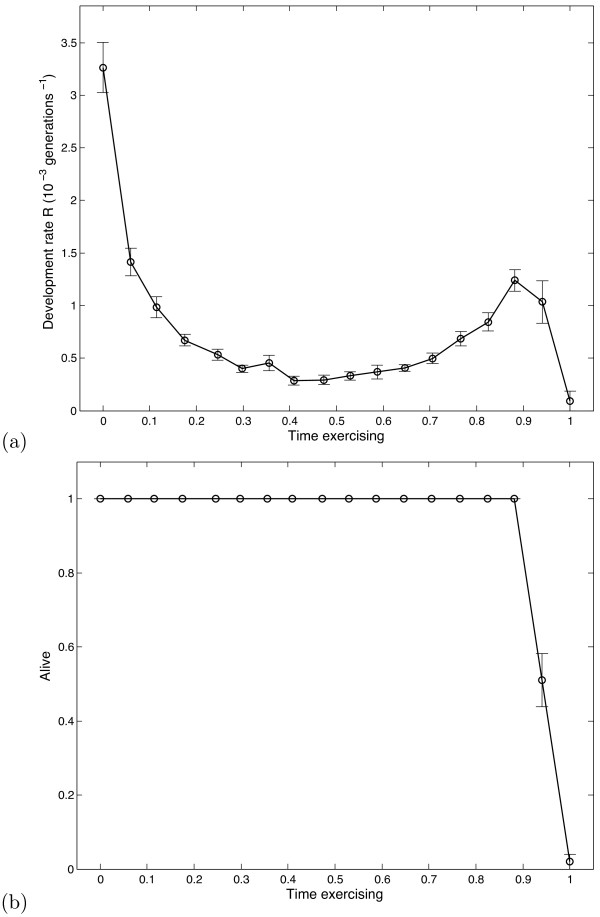
**Effect of transient acidosis**. (a) Variation in the development rate *R *with proportion of time under exercise. Exercise is assumed to correspond to high acidity (*h*_*X *_= 400), whilst during rest acidity drops to normal levels (*h*_*X *_= 0). (b) Variation in epithelium survival with exercise time.

Since the model predicts that permanent acidosis cannot arrest cancer development, we move on to investigate transient acidosis, allowing the system to spend a certain proportion of time at high acidity and a certain proportion at normal acidity; this transient acidosis mimics what occurs when engaging in rigorous exercise followed by rest. In Fig. 3(a) we see how the development rate *R *varies with the amount of time exercising. We see that only a small proportion of time spent at low pH (*h *= 400, a drop of around 0.25 pH units) leads to a significant reduction in *R*. By contrast to the previous figure, the behaviour is not due to total epithelial death (Fig. 3(b)).

Given the discrete and stochastic nature of this model it is not possible to do a complete sensitivity analysis to determine robustness of behaviour to changes in parameter values. However, results from our earlier work [17] show that small changes in parameter values do not markedly change system dynamics. However, that study did indicate that increases in glycolytic rate *k *and adaptation rate *p*_*a *_will generally increase the rate of cancer development, whilst small changes in acid resistance *h*_*T *_have little effect.

Our results support the general concept that transient systemic acidosis can reduce the risk for breast cancer by reducing the rate of somatic evolution. This hypothesis will be explored further with a detailed study of the model.

## Discussion

Breast cancer is the second leading cause of cancer death among women in the United States. In 2005, 186,000 women were diagnosed with breast cancer and 41,000 died of the disease. There are a number of factors that both increase and decrease the risk of breast cancer. Increased physical activity appears to be one of the most consistent and effective strategies for breast cancer prevention. Women who exercise regularly can reduce their breast cancer risk by about 50%. The mechanism of cancer protection is unknown although many hypotheses exist including exercise-induced alteration of ovarian function, weight reduction, or improved immune status.

Recently the authors have proposed that evolution of breast cancer is substantially influenced by regional development of acidosis and hypoxia within ductal carcinoma *in situ *(DCIS). As outlined above, cellular adaptations to these microenvironmental selection forces result in a phenotype that possesses constitutively upregulated glycolysis and resistance to acid-mediated toxicity. This population has a profound proliferative advantage because it can create an acidic microenvironment (through upregulated glycolysis) that is toxic to its competitors but not its own cells.

This model of carcinogenesis suggested an alternative hypothesis for the observed benefit in reducing breast cancer risk. Our hypothesis is based on observations that even moderate exercise can produce significant reduction in blood pH. Thus, we propose that repeated, transient reductions in blood pH due to regular exercise will alter the microenvironmental dynamics of DCIS and that this will be sufficient to prevent or delay its final transition to invasive phenotype.

To examine the feasibility of this hypothesis we use a hybrid discrete cell-based approach to model the evolutionary dynamics of carcinogenesis. The multistep process of carcinogenesis is often described as "somatic evolution" because it appears formally analogous to Darwinian processes wherein phenotypic properties are retained or lost depending on their contribution to individual fitness. According to this model, traits found in invasive cancers must arise as adaptive mechanisms to environmental proliferative constraints during carcinogenesis. In the model presented here we focus on the role of regional variations in microenvironmental selection forces due to decreasing oxygen and glucose concentrations and increasing H^+ ^concentrations that occur with increasing distance from the basement membrane. Carcinogenesis is simplified because mutations in oncogenes and tumour suppressor genes, which constitute multiple independent steps, are lumped into a single proliferation term. Furthermore, we assume a 2D spatial configuration while the true anatomy of a breast duct is a 3D tube-like structure. Nevertheless, we feel that the modified CA model is sufficient to estimate the effects of transient systemic acidosis in the microenvironmental substrate/metabolite concentrations and phenotypic evolution of ductal carcinoma *in situ*.

In comparison to earlier modelling work, we have introduced transient, oscillatory behaviour to mimic the effects of regular exercise. Differences between sustained (Fig. 2) and transient (Fig. 3) acidosis are marked; the results of our initial modelling suggest that repeated transient reductions in intra-tumoral pH_e _will produce significant effects in the evolutionary dynamics of DCIS. It remains to be shown that transient decreases in blood pH would decrease the pH_e _of *in situ *cancers. If this is the case, then our results indicate that the reduction in the incidence of breast cancer in women who undertake increased physical activity could be the result of transient acidosis that increases the time required for DCIS to progress to invasive cancer. Based on this we conclude that additional investigation is warranted.

## Competing interests

The authors declare that they have no competing interests.

## Authors' contributions

KS performed the simulations. All authors drafted the manuscript.

## Reviewers' comments

Natalia Komarova - komarova@math.uci.edu

"Episodic, transient systemic acidosis delays evolution of the malignant phenotype: Possible mechanism for cancer prevention by increased physical activity", describes a new mechanism by which exercise can reduce the risk of cancer. The author's hypothesis is that exercise increases (temporarily) the cellular acidity level, and this leads to a reduction in the rate of carcinogenesis. Numerical simulations based on a hybrid cancer model demonstrate this effect.

The paper is very well written, the idea is novel and interesting (although I am absolutely not qualified to comment on the biological effects of physical activity and its connection with the extracellular acidity level). The effect described by the authors is striking: if the acidity level is reduced permanently, this does not decrease the carcinogenesis rate (up to a point, where all cells die because of the acidity). However, if the acidity level is raised transiently, there is a significant reduction of carcinogenesis level (and there is even a nice optimal amount of transient acidosis, after which things get worse again!).

The authors state that at this moment they do not have a theory of this effect. Creating such a theory (or an intuitive explanation) may be a difficult task. However, to make the present paper with preliminary results more complete, I would suggest to perform some sort of a robustness test. What is the observed phenomenon holds only for the one parameter combination which was explored? How does it change with parameters? Which parameters is it most sensitive to? Apart from making the present paper more convincing, this could indicate the mechanism responsible for the observed behavior.

To conclude, I think this is a very interesting paper and it should definitely be published, once the authors include a robustness/sensitivity analysis of their findings.

Response

• Suggests that we perform a robustness/sensitivity analysis of our findings.

Given the discrete and stochastic nature of this model it is impossible to do a complete sensitivity analysis. We do, however, now comment on the effect of parameter changes in the manuscript.

Heiko Enderling - heiko.enderling@tufts.edu

The authors present a very interesting hypothesis to explain how physical activity can prevent cancer development. The study is based on a verified model of somatic evolution of malignant cell phenotypes in a breast duct. One rate-limiting step in the carcinogenesis step is adaptation to acidosis - an increased level of acidity due to increased glucose consumption as oxygen delivery decreases. The authors introduce increased transient acidity as a consequence of physical activity, which in turn triggers additional cell death. Simulation results show that permanent levels of high acidosis prevent tumor development because normal epithelium dies before cancer can develop. In contrast, transient acidity, as for example a result of physical activity, decreases tumor development rates without destroying normal cells. The simple model supports the proposed hypothesis that transient systemic acidosis can prevent cancer, and the authors correctly point out that further research is necessary to identify the underlying mechanisms.

Suggestions:

• Is the reversibility of phenotypic changes necessary to observe the discussed dynamics? How does the system behave if carcinogenic events are irreversible?

• Figure 3 shows interesting tumor development rates as a function of exercising time. Elaborate on the non-monotonic behavior: why does cancer development decrease as time approaches 0.5, followed by an increase in development rate up to 0.9?

• Weaken the last paragraph of the discussion. "results ... indicate that transient systemic acidosis will produce significant effects in the microenvironment...". Two of the biological questions raised in the background section (p.4) are: "do transient blood pH decrease the pHe of in situ cancers" and "are intratumoral pHe dependent on the blood pH value"? these questions are not answered in this study, and the results do not indicate a change in the environment. The model assumes that there will be a systemic change, and hence the evolutionary dynamics are perturbed. Revise.

Response

• Questions whether reversibility is necessary to observe these dynamics.

We have added a comment on this point to the manuscript.

• Asks why figure 3 shows this non-monotonic behaviour.

It is not clear why there is a non-monotonic response. The reason is likely very complex and we would not like to speculate in the manuscript itself, rather the question will be pursued in further work.

• Suggests we weaken the conclusions drawn in the final paragraph.

We agree with the suggestion, the paragraph now draws a relationship between dynamics and pHe, rather than serum pH as originally stated.

Mark Little - mark.little@imperial.ac.uk

General comments

This is a generally well-written paper, describing a schematic 2D breast cancer model that is used to predict the effects of exercise-induced acidosis. My only major comment is that the parameters used (Table 1) appear to be arbitrary. In particular, to take seriously the predictions made by the model it would be good to know just how tumour cell fitness varies with acidosis.

Specific comments

Is a pH change of this magnitude (6.8) plausible? pH is physiologically very tightly regulated, and I suspect one would very rarely see pH at this level.

Response

• Queries the parameter values used in Table 1.

We now provide a reference in which their provenance is described.

• Asks if an extracellular pH of 6.8 is (physiologically) reasonable.

References are given for both use of this threshold and its physiological relevance.

Yang Kuang - kuang@asu.edu

This is a very interesting and timely paper. The authors argue that their results indicate that the reduction in the incidence of breast cancer in women who undertake increased physical activity could be the result of transient acidosis that increases the time required for ductal carcinoma in situ (DCIS) to progress to invasive cancer. It is a continuation of the authors' recent work [7,14].

To make it more readable, it will be helpful if the authors can provide references to support some of their key statements. For examples, in the second paragraph of background section, the authors stated that using mathematical models, in vitro experiments, and clinical observations, it has been shown that proliferation of in situ cancers carries the cells further away from the basement membrane and, therefore, increasingly distant from the underlying blood supply. It will be necessary to provide references for the mentioned mathematical models, in vitro experiments, and clinical observations. Similarly, references are needed to support other statements in that paragraph (The consequent upregulation of glycolysis combined with long diffusion distances for metabolites produces marked acidosis in the hypoxic regions (need refs). It has been proposed that adaptation to hypoxia is critical in emergence of the malignant phenotype because the combination of adaptations with upregulation of glycolytic metabolism and resistance to acid-mediated toxicity confers a profound proliferative advantage (need refs)).

In particular, even though the authors may have substantiated their model assumptions elsewhere, the authors shall at least cite references to explain their model (1)-(9) assumptions and presenting some minimum model formulation details. It is also necessary to explain the estimation and/or selection of their model parameter values. Table 1 lists model non-dimensional parameter values. It will be useful if the authors can include the parameter units. It will also be helpful if the authors can comment on how much readers shall read into their results given that they are generated from a model with a single set of ad hoc parameter values.

More naturally, the authors may want to compare their new model dynamics with their earlier results [7,14] or highlight the differences and explain the importance of such differences.

Response

• Requests more references to support statements on previous (modelling, experimental and clinical) work.

New references have been included as suggested.

• Asks for further justification of model formulation and results.

We have expanded points of the methods section to clarify and justify modelling assumptions.
